# Infusion-related side-effects during convection enhanced delivery for brainstem-diffuse midline glioma/diffuse intrinsic pontine glioma

**DOI:** 10.1007/s11060-022-04077-6

**Published:** 2022-08-06

**Authors:** Milo Hollingworth, Stergios Zacharoulis

**Affiliations:** 1 Department of Neurosurgery, Queens Medical Centre, Nottingham University Hospital NHS Trust, Nottingham, NG7 2UH UK; 2 Precision Imaging Beacon, School of Medicine, University of Nottingham, Nottingham, NG7 2UH UK; 3 Paediatric Haemato-Oncology, CUMC/Herbert Irving Pavilion, New York, NY 10032 USA

**Keywords:** DIPG, Midline Diffuse Glioma, Convection Enhanced Delivery, Neurological Assessment, Clinical Scales

## Abstract

**Introduction:**

Side-effects during convection enhanced delivery (CED) are poorly understood. We intended to determine the frequency of side-effects during brain stem infusion and determine risk factors for side-effects persisting longer than 24 h.

**Methods:**

Children with a radiological diagnosis of brain stem diffuse midline glioma/Diffuse Intrinsic Pontine Glioma were treated on compassionate grounds with awake infusion of carboplatin and sodium valproate into the brain stem using the 4-catheter (2 trans-cerebellar 2 trans-frontal) chronic, intermittent Renishaw Drug Delivery System. We used change in the Pontine Neurological Observation Score (PONScore), a standardised neurological assessment tool, to identify side-effects during infusion. Recovery was determined by retrospective chart review.

**Results:**

55 infusions were performed in 8 children (3–11 years). Mean PONScore increased during infusion from 3.3 to 5.7 (p-value > 0.001). One hundred and fifty-seven infusion-related side-effects were identified including headache (33/157) and limb weakness (49/157). Fifty-four side-effects persisted > 24 h. Side-effects that had occurred during a previous infusion and those that occurred during infusion via trans-cerebellar catheters were more likely to be persistent with OR 2.333 (95% CI 1.094–4.976; p-value = 0.028) and 2.155 (1.029–4.513; p-value = 0.042) respectively. If infusion was stopped or titrated at onset rather than continued, the side-effect was less likely to persist > 24 h, OR 0.473 (95% CI 0.177–0.948; p-value = 0.037). Most side-effects developed within the first three millilitre of infusion.

**Conclusions:**

Side-effects during brainstem infusion are common, can be transient or persist longer than 24 h. Neurological injury during infusion may be time dependent and accumulative rather than volume dependent.

**Supplementary Information:**

The online version contains supplementary material available at 10.1007/s11060-022-04077-6.

## Introduction

Convection enhanced Delivery (CED) is a promising treatment for Brainstem Diffuse Midline Glioma (BS-DMG; previously Diffuse Intrinsic Pontine Glioma) [1]. In CED one or more catheters are surgically implanted and, by continuous positive pressure infusion, drug is directly infused into the brain parenchyma—bypassing the blood brain barrier (BBB) achieving high intra-tumoural concentrations [2, 3]. This has particular theoretical advantages for treatment of BS-DMG, which is an unresectable lethal childhood brain tumour. BS-DMG is characterised by histone mutations leading to altered H3K27 function and widespread epigenetic dysregulation. Patients between 4 and 11 years present with progressive cerebellar signs, cranial neuropathy and ataxia, with imaging demonstrating expansion of the pons, often in the absence of contrast enhancement. Diagnosis occurs with 4–6 weeks of first symptom and has a median survival of 9 months, characterised by progressive neurological disability. Chemotherapy trials have failed for over 30 years. Radiotherapy remains the standard of care.

As such, CED is an exciting potential therapy for BS-DMG because of its ability to bypass the BBB and deliver chemotherapy to clinically relevant brain volumes. Several trials are underway (NCT03086616; NCT03566199; NCT01502917). In a landmark Phase I dose escalation study of CED in BS-DMG/DIPG,  124I radioisotope was conjugated to a tumour-targeting antibody and delivered to 28 children [2] This study demonstrated that CED could achieve high intra-lesional dosing with negligible systemic exposure, and was well tolerated. Toxicity was defined according to the Criteria for Terminology Common Adverse Events (CTCAE). In this non-randomised study, many of the adverse events may not have been CED-related and could be attributed to concomitant use of corticosteroids, such as hyperglycaemia, neutrophilia and lymphopenia. However, 25% of adverse events were neurological, suggesting local delivery of drugs causes local neurological side-effects. Szychot et al., 2021 described their experience of treating 13 patients on compassionate grounds with carboplatin and sodium valproate using the 4-catheter chronic, intermittent Renishaw Drug Delivery System [3]. The most common reported side effects were mild-moderate headache, transient facial weakness, dysarthria, ataxia, and/or unilateral weakness during infusion. Deficits were described as grade 1–2 mostly returning to pre-infusion baseline within 24 h of stopping infusion.

Quantification of toxicity in CED and BS-DMG is complex. Toxicity in CED can arise due to: (1) the effect of catheter implantation; (2) the infusion; or (3) the pharmacological action of the drug. The new or worsening neurological deficits occurring during infusion often improve after the infusion is stopped. However, the cause and recovery of these deficits can be difficult to interpret. When infusion is stopped, local drug concentrations remain high. The patient’s clinical phenotype is superimposed on aftereffects of surgery (catheter implantation and/or biopsy) and may occur at different stages of a rapidly progressive disease. As such, neurological changes in BS-DMG patients occurring during CED may come and go, may persist, become permanent or even progress. Furthermore, neurological dysfunction cannot always be easily elicited in children; relying on subjective, and often opportunistic, examination. This is also compounded by the difficulties of performing randomised controlled trials of CED in BS-DMG, which include the requirement of supra-specialist facilities to deliver treatment, the rarity of the disease, the absence of good treatment options and the availability of alternative, and often well-publicized, experimental treatments inside and outside of clinical trials. Understanding causality in CED-related toxicity is therefore very challenging, requiring careful thought about how best to understand and reduce the side-effects of this emerging treatment. Szychot et al., 2021 described a novel patient evaluation system used to monitor side-effects during infusion. This patient evaluation system uses a standardised neurological assessment that elicited signs and symptoms of headache, ophthalmoplegia, bulbar dysfunction, paraesthesia, limb weakness and cerebellar dysfunction and amalgamated them into a numerical score, with increasing scores reflecting increasing disability [3]. This score, the Pontine Observational Neurology Score (PONScore) was found to have excellent intra-rater reliability that increased during infusion to reflect the accumulation of neurological signs and symptoms during infusion [4]. We hypothesised that the PONScore could be used to determine the frequency of side-effects during infusion, and this would help better understand mechanisms of toxicity and likelihood of recovery.

## Methods

### Ethical approval

Treatment of patients using CED on compassionate grounds was approved by the institutional ethics committee and was compliant with the 1964 Helsinki declaration and its later amendments. Implantation of the drug delivery system was approved by the Medicines and Healthcare Regulatory Authority, United Kingdom. Parents were consented for the experimental nature of the treatment and the use of their child’s information for the development of the treatment and for scientific publication. Data was acquired from routinely collected information as part of their clinical treatment.

### Pontine infusion

**Fig. 1:**
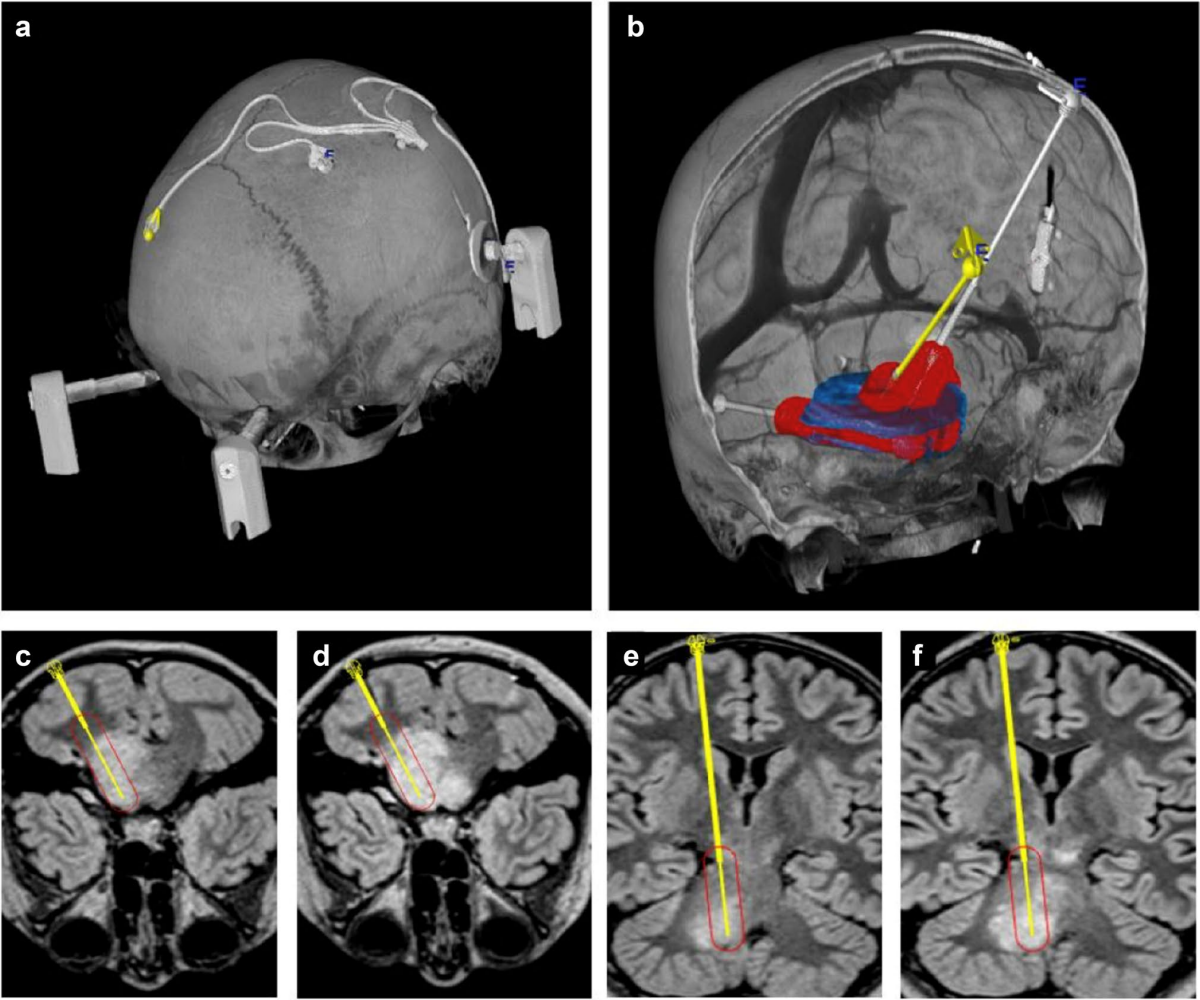
Treatment of the diffuse intrinsic pontine glioma using a chronic, implantable Renishaw Drug Delivery System. Three-dimensional reconstructed computer tomography of a four- catheter drug delivery system is shown in situ (**a**). Recessed stepped catheters provide controlled reflux (red) along the distal catheter trajectory targeting the tumour (blue) (**b**). Fluid-attenuated inversion recovery sequences of the left cerebellar and frontal catheters before (**c**, **e**) and after (**d**, **f**) infusion of 4.1 mL of chemotherapy showing distribution of drug

The technique of CED described herein has been described in detail [3]. Catheters were positioned to maximise tumour coverage during infusion, avoid cystic regions or hemorrhage. Trajectories would be planned to avoid venous sinuses, ventricles and vessels based on a co-registered computer tomography angiogram. Exact positions differed between patients; however, catheters had similar positions between patients. Trans-cerebellar catheters traversed the cerebellar hemispheres and terminated in the lateral anterior pons via the cerebellar peduncles. Trans-frontal catheters traversed either the anterior or posterior tegmentum, depending on individual anatomy, and terminated at the ponto-medullary junction. These catheters were connected to a bone-anchored transcutaneous drug administration port via sub-galeal tubing (Renishaw Drug Delivery System; Renishaw Plc, Wooton-under-Edge, UK). Following implantation, patients would be recovered from anaesthetic and commence infusion within 72 h of implantation. Infusion would be conducted over two days, the first day infusion would be conducted via trans-frontal catheters and via trans-cerebellar catheters on the second day. This strategy was employed to maximise cumulative infusion volume over the two-day cycle. Patients would sit in bed connected to an infusion set connected by two-metre-long extension lines. Infusion would be commenced at 0.03 ml/min/catheter for 10 min and increased by 0.03 ml/min/catheter every 5 min until a maximum of 0.18 ml/min/catheter was reached; these values as well as infusion concentration were developed from prior clinical experience and large animal models [5–7]. Infusions would be continued until the onset of neurological symptoms to maximise the volume of tumour treated. Neurological symptoms were elicited by the attending medical team using clinical assessment. Based on prior experience [5–7], deficits were deemed transient and as such, if a deficit could be localised the flow rate would be reduced by 50%. If the deficit failed to improve, was moderate to severe, or could not be localised, the infusion was stopped. The final volume infusion volume would depend on the patient side-effect profile and tolerance. Each treatment would typically consist of two infusions over two separate days. This international cohort of patients treated on compassionate grounds would be treated at 3–8 weekly intervals depending on patient fitness, personal arrangements with other health care providers, and memory-making experiences. (Fig. 1).

### The PONScore

The development and validation of the PONScore have previously been described [4]. In brief, the PONScore is a 57-point scale that elicits the signs and symptoms of brainstem dysfunction arising during pontine infusion, with increasing scores reflecting greater degrees of severity.

### Data analysis

Data recorded during the routine care of patients was collected retrospectively from 55 pontine infusions. PONScores were measured by the attending Paediatric Intensive Care Unit (PICU) nursing staff before infusion initiation and at every hour during infusion until the end. PONScores were inputted directly onto a computer database using critical care and anaesthesia information software, ICIP® (Phillips, Surrey), together with the infusion rates and volumes per catheter. Infusion-related side-effects were identified by increases in the PONScore. Side-effects detected using the PONScore were compared to the case notes. If a new side-effect was identified during infusion by an increase in the PONScore and the deficit was documented in the patient chart (according to parent and patient interview, review of outpatient letters or clinical examination) more than 24 h after the end of the infusion, the side-effect was defined as a persistent infusion-related side-effect. Transient infusion-related side-effects were defined as side-effects identified during infusion that were not present > 24 h following infusion. Data was analysed using Statistical Package for Social Science (Version 23; IBM, USA). Comparisons between PONScores were performed using *t*-test calculation. Correlation calculations were calculated using Spearman’s correlation co-efficient. Frequency of side-effects were determined at the time of onset. Infusion related side-effects were dichotomised into transient and persistent and logistic regression was performed to determine odds ratios (OR) for persistent side-effects. Statistical significance was defined as p-value < 0.05.

## Results

**Fig. 2:**
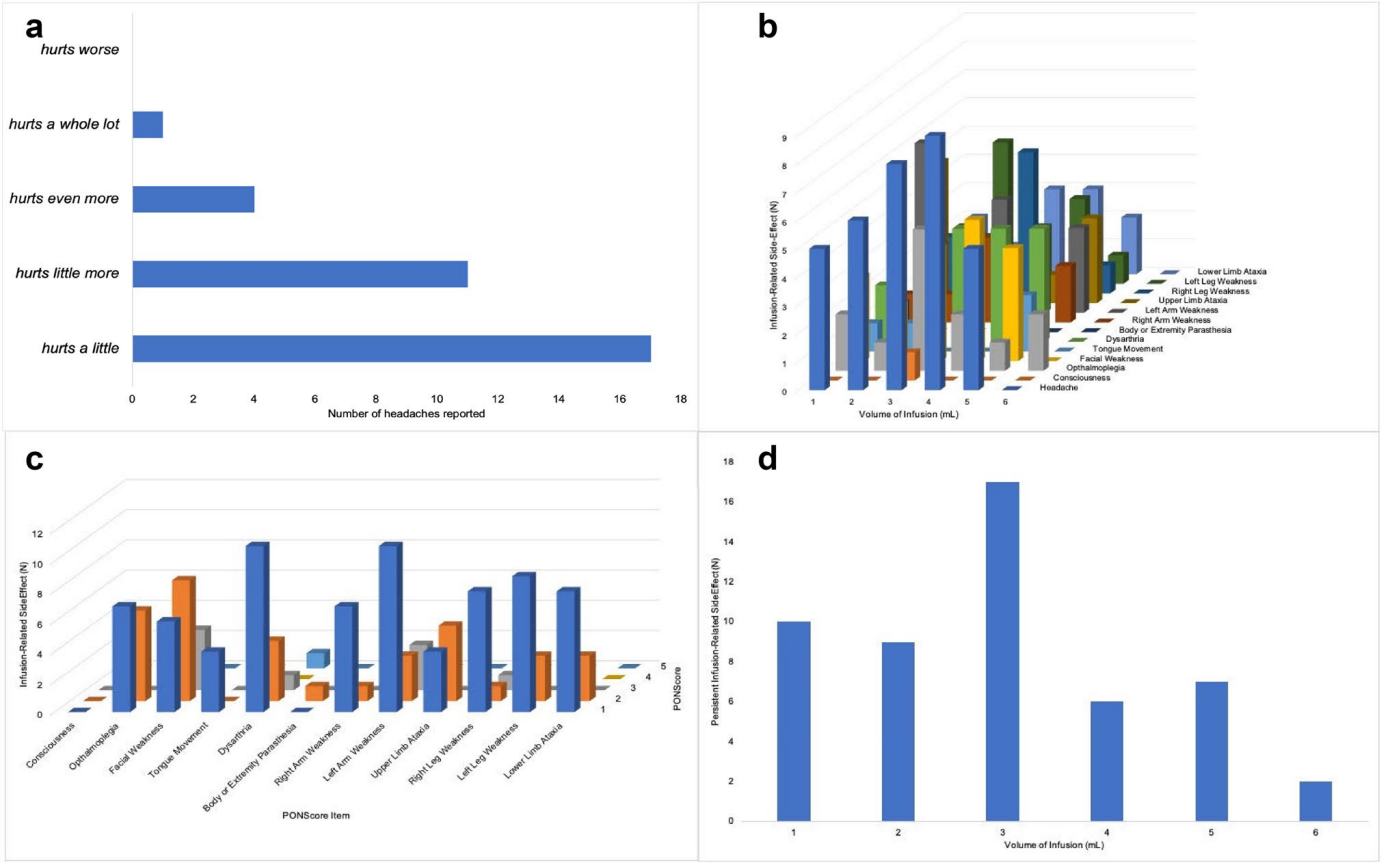
Frequency and severity of infusion-related side-effects during pontine infusion measured using change in PONScore from pre-infusion baseline. Headache was reported during pontine infusion, and severity was recorded using the WONG Baker Faces Scale (**a**). An array of neurological signs and symptoms were detected during infusion using the PONSscore, mostly resulting in an increase by 1–2 points (**b**, **c**). Most persistent Infusion-Related Side-Effects occurred in the first 3 mL of infusion

**Fig. 3:**
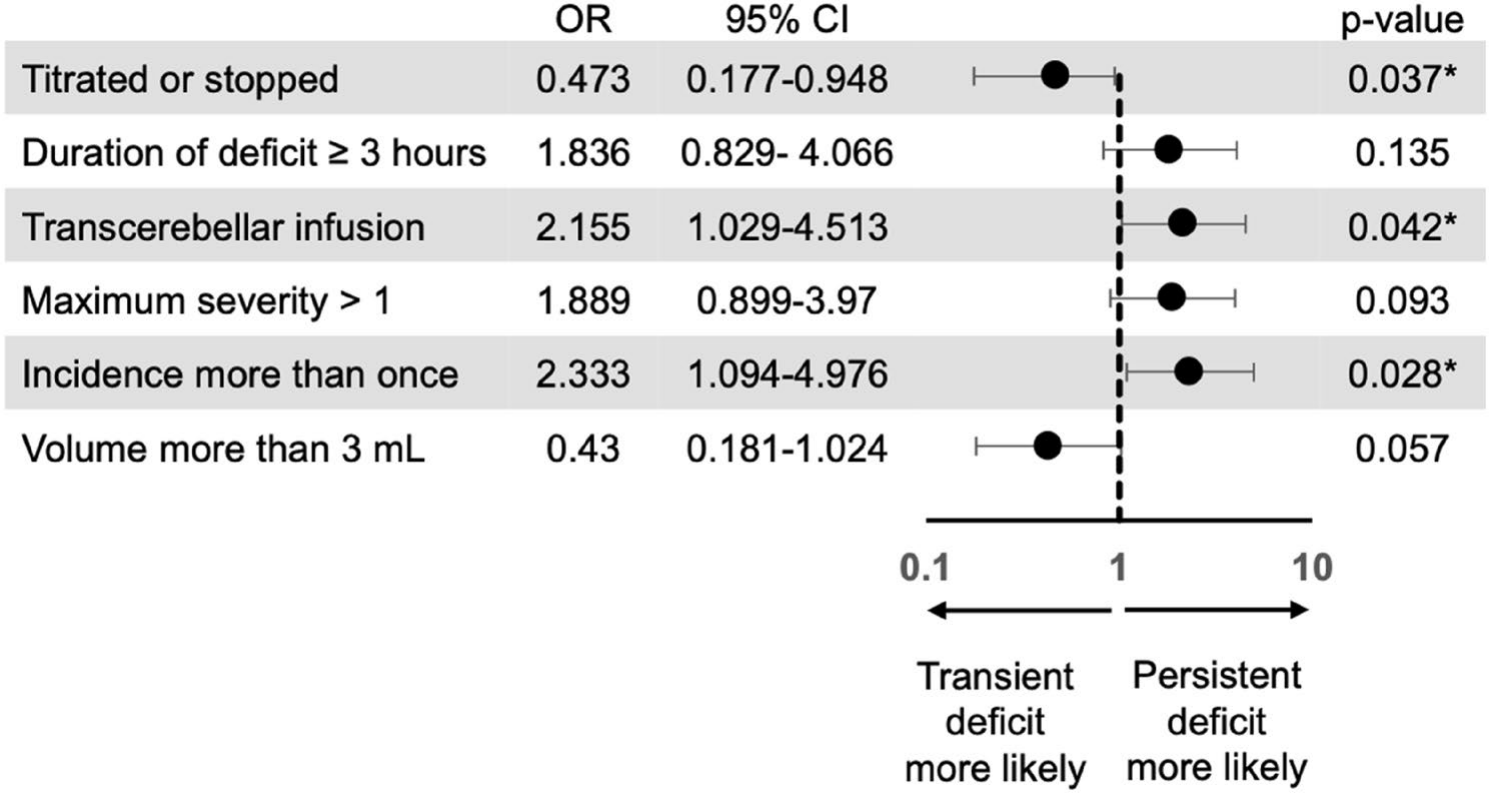
Risk factors for persistent deficits arising from 55 pontine infusions in eight children with BS-DMG/DIPG. A forest plot demonstrating unadjusted odds ratios for risk of an infusion-related deficit becoming persistent

Fifty five infusions of carboplatin combined with sodium valproate were studied in eighty children (3–11 years; Table 1). These infusions represent the earlier stages of the Sychot Cohort and their characteristics have previously been described [3]. Median PONScore at the start of each infusion was 2 (range 0–16). Pre-infusion baseline PONScores demonstrated weak (0.45) and moderate (0.63) correlations with numbers of infusions received and days since diagnosis respectively using Spearman correlation co-efficient calculations, both of which reached statistical significance (p-value < 0.001). Mean PONScore increased during infusion from 3.3 to 5.7, which differed with statistical significance (p-value > 0.001). PONScores identified 157 infusion-related side-effects during 55 pontine infusions, the most common of which were headache (33/157) and limb weakness (49/157) (Fig. 2). The assessment of headache severity, which was derived from the Wong Baker Faces Scale, reported as hurting ‘a little’, ‘a little more’, ‘even more’ and ‘a whole lot’ in 17, 11, 4 and 1 cases respectively. (Fig. 2). Side-effects ranged from cranial neuropathies, limb weakness and cerebellar signs, which occurred throughout infusion (Fig. 2). The severity of change was usually only mild, indicated by an increase of one or two points of the PONScore (Fig. 2). Transient and persistent neurological deficits were identified respectively in 70 and 54 cases respectively. Deficits that had occurred during a previous infusion and those that occurred during a trans-cerebellar infusion were more likely to be persistent with OR 2.333 (95% CI 1.094–4.976; p-value = 0.028) and 2.155 (1.029–4.513; p-value = 0.042) respectively. If an infusion was stopped or titrated rather than continued, the deficit was less likely to be persistent, OR 0.473 (95% CI 0.177–0.948; p-value = 0.037). Other factors such as maximum severity of deficit, increase in PONScore by > 1, and duration of deficit during infusion longer than 3 h trended toward increased risk of persistent deficit but failed to reach statistical significance. Deficits acquired after 3 mL of infusion tended toward reduced risk of becoming persistent; however, this did not reach statistical significance (Fig. 3).

**Table 1:** Eight children with DIPG/BS-DMG receiving infusion of carboplatin and sodium valproate by intermittent, chronic Convection Enhanced Delivery using the Renishaw Drug Delivery System

Case	Age at diagnosis (y)	Biopsy	Diagnosis to Implant (months)	Number of pontine infusions studied
1	10.6	–	4.9	10
2	7.8	–	7.9	1
3	6.9	–	3.9	8
4	6.3	H3K27M	3.3	4
5	10.7	H3K27M	4.2	12
6	6	H3K27M	4.9	6
7	3.6	H3K27M	3.6	6
8	5.7	–	6.5	8

## Discussion

Neurological signs and symptoms in eight children during 55 pontine infusions of combined carboplatin and sodium valproate were systematically monitored using the PONScore. The PONScore increased during pontine infusion reflecting the accumulation of neurological signs and symptoms during infusion. The most common infusion-related side-effects were headache and limb weakness. Headache was mostly mild to moderate, while every patient developed at least a sign or symptoms of neurological dysfunction. 50/124 cases of infusion related side-effects identified using the PONScore persisted for > 24 h. Important predictors of persistent side-effects were recurrence of the side-effect during a previous infusion, infusion through trans-cerebellar catheters and continuation of infusion at the onset of side-effects, rather than stopping or reducing the rate of infusion.

There are several limitations to this analysis and the extent to which its results can be generalised. Patients were treated on compassionate grounds and underwent surgery following recovery from radiation treatment. As such, these results may not be born out in a prospective clinical trial or in children with a greater burden of pre-treatment disability. Patients were also receiving other therapies alongside their CED treatment, and this may have contributed to their side-effect profiles. The retrospective analysis has inherent limitations, and risk factors for persistent deficits should be interrogated in prospective studies. This study also reports the side-effects of a highly specialist treatment, only performed in a handful of international centres, and hence may not be relevant to other treatments of DIPG/BS-DMG. However, the study should serve as a guide for those caring for patients, or for those considering or receiving this therapy and to our knowledge is the first publication to describe infusion-related side-effects in detail. As the PONScore is in its developmental infancy, further studies are required to determine its reliability and validity as an outcome measure and its normative values are unknown. Nursing staff who performed the assessment were unblinded to the infusion volumes and parameters. There is also no understanding of what change in PONScore is clinically significant, and many detected side-effects may not reach a clinically meaningful threshold. The definition of recovery used retrospective chart review, and future prospective studies should consider using the same standardised neurological assessment tool, such as the PONScore, to quantify recovery. In addition, side-effects were related to anatomical location, and therefore catheters in different locations may not reproduce the same side-effects. Due to differences in the number of infusions received prior to disease progression, infusions studied were also not equally divided between patients; and hence observed findings are skewed toward the patients who received the most infusions. However, despite these limitiations, this is the first attempt to use a standardised neurological assessment tool to describe the neurological changes during brain stem infusion and it makes important observations about the mechanisms of infusion related toxicity.

The description of symptomatology during infusion are corroborated by Souweidane’s et al., 2019 who describe a similar array of cranial neuropathies, limb weaknesses and long tract signs after brainstem infusion [2]. We cannot make comparisons regarding the frequency and severity of side-effects because Souweidene et al,. defined toxicity using CTCAE at different time-points relative to infusion [2]. Szyshot et al., 2021 used clinical assessment to describe toxicity. Our findings support their conclusion that neurological side-effect mostly resolved within 24 h [3]. However, by using an increase in PONScore to identify side-effects, it appears a significant minority of infusion-related side-effects persist for longer than 24 h. It is imperative that infusion-related side-effects are meticulously documented and their mechanisms understood.

**Fig. 4:**
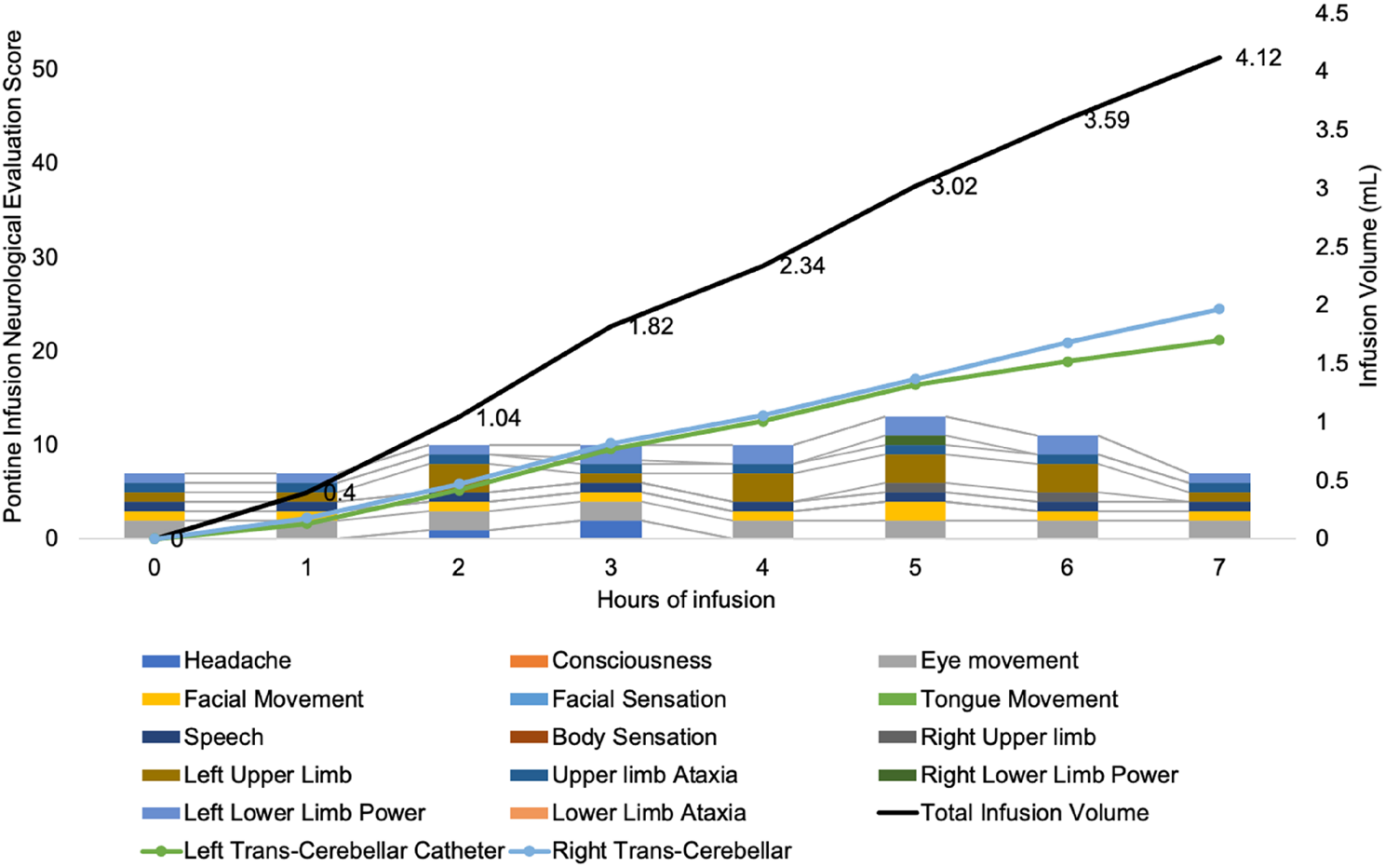
An Infusion Profile. During infusion the patient acquires neurological signs and symptoms indicated by an increase in PONScore. At hour 5, after infusion of 3.02 mL, there is evidence of worsening facial movement, right arm and leg power. Flow rate down the left cerebellar catheter is reduced, after which there is a reduction in PONScore

There are several factors that could influence the development (and detection) of side-effects during an infusion. Patient factors such as age, pre-infusion clinical status and the anatomy of their tumour may impact the ability to detect or tolerate infusion. Indeed, the anatomical position of the catheters, mechanical effect of the infusion or pharmacological toxicity represent modifiable factors that could lead to toxicity. Without careful study in appropriately designed clinical trials using well designed clinical tools, answering these questions is challenging. Our findings make important contributions to understanding these phenomena. We demonstrate that side-effects frequently occur during infusion. It has been previously described how side-effects improve following cessation of infusion [3]. Taken together, it may be that the major source of infusion-related toxicity is due to the mechanical effects of infusion rather than direct pharmacotoxicity. Side-effects were more likely to become persistent if the infusion was continued rather than reduced or stopped at symptom onset. At the point of flow rate adjustment, local drug concentrations within the core of the distribution volume will remain stable up to 12–24 h after adjustment [7]. The association between flow rate reduction and improved recovery suggests that infusion related side-effects are due to mechanical effects of infusion. Although, the effects of pontine infusion on interstitial pressure in treatment of BS-DMG are lacking, it is reasonable to assume that as infusion rate is reduced, interstitial pressure within the pons will also fall, or stabilise. This may explain how side-effects that occurred during infusion improved following adjustment of flow rate (Fig. 4). It may be that infusion leads to increased interstitial pressure, which ultimately impairs locoregional perfusion. This may explain the variability in side-effects between patients. BS-DMG/DIPG are characterised by non-uniform perfusion, brought about by a hypercellular cytoarchitecture with irregular capillary networks (8). Catheters in the same locations within the pons may elicit different side effects depending on the unique interactions between the effect of applied infusion pressures on local tumoural perfusion.

We also found that if an infusion related side-effect was observed during a previous infusion it is more likely to become persistent. This suggests infusion-related neurological injury is accumulative, and therefore reducing the risk of infusion-related side-effects is central to the future of the therapy. This theory is confounded by the interaction between numbers of infusions and time from diagnosis. However, considering that the infusions studied were performed prior to diagnosis of clinical and radiological progression, it is likely that infusion is an important contributory factor to the accumulation of neurological disability over time. Side-effects were also more likely to be persistent if acquired during infusion via trans-cerebellar versus trans-cerebral trajectories. Trans-cerebellar infusions were typically conducted on the second day of infusion, therefore, greater risk of side-effects may be due to the accumulative injury from the previous day. It could also be due to the anatomical arrangement of the catheters. Transcerebellar catheters are placed into the anterolateral pons containing corticospinal tracts and crossing ponto-cerebellar fibres, which may be more sensitive to distortion. High resolution tractography and perfusion mapping may help to direct catheters away from brain stem nuclei and tracts and critically ischemic tumour regions.

It is evident that infusion-related side-effects were not directly correlated to infusion volume. It is evident that 30% of the recorded deficits occurred < 1 mL infusion. Interestingly, fewer side-effects occurring at the end of infusion became persistent. The risk of persistent side-effects was increased if the infusion was continued (rather than stopped or reduced) and there was a non-significant tendency toward greater risk of persistent side-effects if the infusion was continued for ≥ 3 h after deficit onset. This would suggest that the way in which the infusion is conducted is a key determinant of treatment-related side-effect profile. Performing the infusions awake, in such a way that patients can be constantly monitored and flow rates adjusted may be important avenues to achieve large volume infusions. Slowing down the ramping regime at the beginning of the infusion may also reduce critical pressures that could lead to infusion-related side-effects.

Regardless of the underlying cause of these side-effects, many of these hypotheses can only be tested in human subjects. As described, the neurological phenotype arising during pontine infusion is likely due to the interaction of catheter location, the morphology of fluid distribution and pathology of the underlying tumour, and the pharmacology of the drug. A reliable means of quantifying neurological change would be hard to achieve in an animal model, even with expert veterinary supervision. Without standardised neurological examination, addressing such questions will be impossible in human studies. In summary, we present findings from 55 pontine infusions in eight children with BS-DMG/DIPG. Analysis of neurological signs and symptoms occurring during pontine infusion using the PONScore demonstrated that risk of persistent infusion-related side-effects were higher if the infusion was not immediately reduced and if the side-effect had occurred during prior infusions. If catheter flow was adjusted based on anatomical position some deficits would recover. This suggests that the neurological injury arising from pontine infusion is often localised, time dependent, accumulative rather than volume dependent, and this means they may be ultimately modifiable. The study of interstitial pressure and perfusion and their interaction with neurological function may be central to development of the treatment.

## Supplementary Information

Below is the link to the electronic supplementary material.

Supplementary file1 (DOCX 13 kb)

## Data Availability

Not applicable.
