# Supplementary material for: Infusion-related side-effects during convection enhanced delivery for brainstem-diffuse midline glioma/diffuse intrinsic pontine glioma
Source: J Neurooncol. 2022 Aug 6;159(2):417–24. doi: 10.1007/s11060-022-04077-6 (PMC9424151; doi:10.1007/s11060-022-04077-6)
Supplement: Supplementary file 1 — Supplementary file1 (DOCX 13 kb) [file 11060_2022_4077_MOESM1_ESM.docx]

Supplementary Material 1. Adverse Events detected using the PONS score during pontine infusion of chemotherapeutics in 8 children with DIPG/BS-DMG

|  |  |
| --- | --- |
| Case | Adverse Events occurring during infusion |
| 1 | headache, ophthalmoplegia, facial weakness, tongue weakness, dysarthria, parasthesia, limb weakness, ataxia |
| 2 | headache, facial weakness, tongue weakness, limb weakness |
| 3 | headache, facial weakness, dysarthria, limb weakness, ataxia |
| 4 | headache, ophthalmoplegia, limb weakness, ataxia |
| 5 | ophthalmoplegia, dysarthria, limb weakness, ataxia |
| 6 | ophthalmoplegia, facial weakness, limb weakness, ataxia |
| 7 | ophthalmoplegia, facial weakness, limb weakness, ataxia |
| 8 | headache, ophthalmoplegia, facial weakness, limb weakness, ataxia |
